# Anti-angiogenic potential of an ethanol extract of *Annona atemoya* seeds in vitro and in vivo

**DOI:** 10.1186/1472-6882-14-353

**Published:** 2014-09-23

**Authors:** Jin-Mu Yi, Jong-Shik Park, Jun Lee, Jin Tae Hong, Ok-Sun Bang, No Soo Kim

**Affiliations:** KM-Based Herbal Drug Development Group, Korea Institute of Oriental Medicine, 1672 Yuseong-daero, Yuseong-gu, Daejeon 305-811 Republic of Korea; Department of Korean Medicine Life Science and Technology, Korea University of Science and Technology, Daejeon, Republic of Korea; College of Pharmacy, Chungbuk National University, Cheongju, Republic of Korea

**Keywords:** *Annona atemoya*, Angiogenesis, Anticancer, HIF, VEGF

## Abstract

**Background:**

Angiogenesis, which is initiated by certain tumor micro-environmental conditions and diverse protein factors, plays a pivotal role during tumor development and metastasis. Therefore, many efforts have been made to develop effective anti-angiogenic agents as anticancer therapeutics. In the current study, we investigated the anti-angiogenic potential of an ethanol extract of *Annona atemoya* seeds (EEAA) in vitro and in vivo.

**Methods:**

The anti-angiogenic potential of EEAA was evaluated using various in vitro/in vivo models, including cell proliferation, migration, and tube formation by human umbilical vascular endothelial cells (HUVECs); a Matrigel plug assay; and tumor-induced angiogenesis. The expression of hypoxia-inducible factors (HIFs) and vascular endothelial growth factor (VEGF) was investigated using reverse transcription-polymerase chain reaction, immunoassays, and western blotting.

**Results:**

EEAA was able to significantly inhibit the angiogenic properties of HUVECs in vitro as well as angiogenic factor-induced blood vessel formation in vivo. EEAA down-regulated the expression of VEGF and HIF-1alpha/2alpha at the mRNA and protein levels, respectively, in cancer cells under hypoxic conditions.

**Conclusions:**

EEAA shows a strong anti-angiogenic potential in both in vitro and in vivo systems, and we suggest that EEAA may be a valuable herbal source for anticancer drug development.

## Background

Angiogenesis, the formation of new blood vessels from pre-existing vasculature [1], is a complex cellular process that consists of endothelial cell proliferation, migration, and differentiation and differs from vasculogenesis, which occurs during embryonic development [2]. Angiogenesis is observed during embryonic vascular development, wound healing, and organ regeneration [3]. In adults, angiogenesis is only observed in specific areas, such as the endometrium and ovarian follicle cells [4]. Pathologically, angiogenesis is closely related to diverse diseases, particularly cancer. Angiogenesis promotes tumor growth by continuously supplying nutrients and oxygen to the tumor and removing metabolic wastes produced by tumor cells [5]. In fact, the volume of a tumor cannot exceed >1 mm^3^ in an avascular state [6]. Metastasis is also initiated by tumor angiogenesis. Tumor cells release chemotactic factors such as vascular endothelial growth factor (VEGF), and these pro-angiogenic factors recruit endothelial cells to tumors [7, 8]. Since angiogenesis was first postulated by Folkman in 1971 [9], it has been considered an intriguing target for developing novel anticancer therapeutics. Additionally, many efforts have been dedicated to developing effective inhibitors targeting angiogenesis, such as natural endogenous inhibitors (e.g., angiostatin), endothelial cell growth inhibitors (e.g., TNP-40), neutralizers of pro-angiogenic molecules (e.g., VEGF receptor antibodies), and enzyme inhibitors modulating basement membrane structures (e.g., matrix metallopeptidase inhibitors) [7]. The advantage of anti-angiogenesis as an anticancer target is that it is a common feature of tumors irrespective of their type, and the development of drug resistance by normal endothelial cells is unlikely due to their low mutagenesis rate [6].

Annonaceae is a family composed of more than 130 genera and 2,300 species worldwide, which are almost exclusively tropical trees and shrubs [10]. Powdered seeds and leaves of *Annona squamosa* L. have traditionally been used to treat head and body lice [11]. Additionally, an *A. squamosa* seed extract is toxic to the cabbage looper, *Trichoplusia ni* [12]. *A. cherimola* Mill. seed extract displays a potential for insecticidal use [13]. In 1907, *A. atemoya* was first produced by Wester by crossing *A. squamosa* and *A. cherimola* [10]. Crude extracts of *A. atemoya* seeds can potentially be used in botanical insecticides, and bullatacin, which is an annonaceous acetogenin isolated from *Annona atemoya*, has been reported to induce apoptotic cell death in the human 2.2.15 hepatocarcinoma cell line by reducing intracellular cAMP and cGMP levels [14, 15]. Fu *et al.* reported that the annonaceous acetogenin 89–2, isolated from *A. atemoya*, is cytotoxic to multidrug-resistant (MDR) KBv200 cells and to chemotherapy-sensitive parental KB cells in vitro, and can successfully inhibit the growth of KBv200 and KB xenografts in nude mice [16]. Fu *et al.* further suggested that 89–2 shows the potential to inhibit P-glycoprotein activity in KBv200 cells as a mechanism to overcome MDR. However, the effect of the ethanol extract of *Annona atemoya* seeds (EEAA) on angiogenesis and its underlying mechanism remain unknown. In this study, we demonstrated that EEAA exhibits anti-angiogenic potential both in vitro and in vivo. To our knowledge, this report is the first to demonstrate the anti-angiogenic effect of a crude extract of *A. atemoya* seeds.

## Methods

### Preparation of EEAA

*A. atemoya* seeds were purchased from Hannong Bio Industry (Jeju, Republic of Korea). The identity of the *A. atemoya* seeds was identified by Dr. Go Ya Choi of the Basic Herbal Medicine Research Group, Herbal Medicine Research Division, Korea Institute of Oriental Medicine (KIOM), Daejeon, Republic of Korea. A voucher specimen (KIOM010068) was deposited at the KM-Based Herbal Drug Research Group, Herbal Medicine Research Division, KIOM. Dried *A. atemoya* seeds (200 g) were finely pulverized and immersed in 70% (v/v) ethanol (100 g/L). Solvent extraction was performed by subjecting the mixtures to two cycles of ultrasonication for 1 h. The extracts were filtered through Whatman No.2 filter paper and concentrated in a rotary evaporator. The powdered extract (EEAA, 20.98 g) was homogenized using a mortar and stored at 4°C until use. The yield of the final extract was 10.49% (w/w). The EEAA was dissolved in dimethyl sulfoxide (DMSO, Sigma, St Louis, MO, U.S.A.) at a concentration of 2 mg/mL and stored at −70°C until use.

### Cell culture

Human umbilical vein endothelial cells (HUVECs) were obtained from Lonza (Walkersville, MD, U.S.A.). The HUVECs were maintained in EGM-2 endothelial growth medium (Lonza) supplemented with 2% fetal bovine serum (FBS), 0.4% fibroblast growth factor-2 (FGF2), 0.1% VEGF, 0.1% R3-insulin-like growth factor-1, 0.1% epidermal growth factor, 0.04% hydrocortisone, 0.1% ascorbic acid, 0.1% heparin, and 0.1% GA-100 at 37°C in a humidified atmosphere containing 5% CO_2_. The culture medium was replaced with fresh medium every other day, and the cells were used for experiments only between passage numbers 5 and 10. A human non-small cell lung carcinoma cell line, NCI-H460, was obtained from the American Type Culture Collection (Manassas, VA, U.S.A.) and was maintained in RPMI 1640 medium supplemented with 10% FBS, 100 units/mL penicillin, and 100 *μ*g/mL streptomycin (Invitrogen, Carlsbad, CA, U.S.A.).

### Cell viability

One day before drug treatment, HUVECs were seeded at a density of 5 × 10^4^ cells/well in a 24-well tissue culture plate containing 500 μL of EGM-2. After 24 h of drug treatment, the culture media were saved, and the cells were trypsinized and then resuspended in the retained culture media. The numbers of viable and dead cells were automatically determined using an ADAM-MC automatic cell counter (NanoEnTek, Seoul, Republic of Korea) as previously described [17].

### Cell migration assay

Once HUVEC growth reached confluence, scratches were introduced with a 200 *μ*L yellow pipette tip and photographs were taken using an inverted microscope (Olympus IX71, Tokyo, Japan). The cells were washed with fresh EGM-2 medium and further incubated in fresh EGM-2 medium containing various concentrations of EEAA. After 18 h, photographs were taken, and wound healing was digitally quantified using the MetaMorph image analysis software (Molecular Devices, Downingtown, PA, U.S.A.). The healing area (%) was calculated according to the following formula: healing area (%) = [1-wounded area (t = 12 h)/wounded area (t = 0 h)] × 100.

### Tube formation assay

The formation of three-dimensional tubes by HUVECs was induced using a Cultrex in vitro angiogenesis assay kit (Trevigen, Gaithersburg, MD, U.S.A.) according to the manufacturer’s instructions. HUVECs resuspended in EGM-2 media containing various concentrations of EEAA were added in a 96‒well plate pre-coated with basement membrane extracts (BMEs). After 8 h of cultivation at 37°C, the tubes were photographed using a microscope. The tube length and branch points were digitally quantified using the MetaMorph image analysis software.

### In vivo angiogenesis assay

A directed-in vivo angiogenesis assay kit (Trevigen) was used to investigate the effect of drugs on in vivo angiogenesis induced by pro-angiogenic factors (VEGF and FGF2) as previously described [17]. Briefly, angioreactors pre-filled with growth factor-reduced BME and a combination of VEGF, FGF2, and EEAA were subcutaneously implanted into the dorsal flanks of 6-week-old Balb/cSlc-nu/nu female mice. After 12 days, the implanted angioreactors were removed, and the degree of new vessel formation was determined via FITC-lectin-mediated quantification of vascular endothelial cells infiltrating the angioreactors. Fluorescence intensities were determined using an excitation wavelength of 485 nm and an emission wavelength of 510 nm (SpectraMax Gemini EM, Molecular Devices). This animal study was approved by the Institutional Animal Care and Use Committee of the Korea Institute of Oriental Medicine (Protocol # 12–058).

### Hypoxia-inducible factor (HIF)-luciferase reporter assay

An NIH3T3 stable cell line (NIH3T3/HIF-luc) carrying a hypoxia-responsive element (HRE)-derived luciferase reporter system (pHIF1-Luc) was obtained from Panomics (Fremont, CA, U.S.A.). The NIH3T3/HIF-luc cells were maintained in DMEM basal medium supplemented with 10% FBS, 100 units/mL penicillin, 100 *μ*g/mL streptomycin, and 100 *μ*g/mL hygromycin B (Invitrogen) in 5% CO_2_ humidified air at 37°C. Luciferase expression was induced by exposing the cells to 150 *μ*M of CoCl_2_ or hypoxic gas (1% O_2_, 5% CO_2_, N_2_ balanced). After 24 h of induction under hypoxic conditions, the cells were washed twice with ice-cold PBS, and a whole cell lysate (WCL) was prepared using the 1X passive lysis buffer included with the luciferase assay system (Promega, Madison, WI, U.S.A.). Preparation of the WCL and quantification of intracellular luciferase activities were performed according to the manufacturer’s guide.

### Immunoassay

The quantity of VEGF released from NCI-H460 cells into the culture medium was determined using a human VEGF immunoassay kit (R&D Systems, Minneapolis, MN, U.S.A.) according to the manufacturer’s instructions. Briefly, the culture medium was cleared via centrifugation at 4,000 g for 10 min at 4°C. The recombinant VEGF standards and cleared medium samples were loaded into a 96-well immunoplate pre-coated with a VEGF monoclonal antibody. The samples were incubated for 1 h at room temperature (RT). After 3 washes, a horseradish peroxidase (HRP)-conjugated polyclonal antibody against VEGF was added to each well and incubated for 3 h at RT. After 3 washes, color development was initiated by adding a substrate solution and was monitored at 450 nm using a microplate reader (Emax, Molecular Devices). Extracellular VEGF was quantified based on the standard curve.

### Real-time polymerase chain reaction

TaqMan real-time polymerase chain reaction (RT-PCR) was performed to determine the effect of EEAA on intracellular VEGF mRNA levels. Total RNA was prepared from NCI-H460 cells that had been cultured in the presence or absence of EEAA using the Easy-spin™ total RNA extraction kit (iNtRON biotechnology, Seoul, Republic of Korea). The integrity of the isolated total RNA was confirmed via agarose gel electrophoresis. Single-stranded cDNA was synthesized from 5 *μ*g of total RNA using the SuperScript™ III first strand synthesis system (Invitrogen). The pre-validated probe and primer sets for VEGF [RefSeq, NM_001025366.2; ABI ID, Hs00900055_m1] and β-actin [RefSeq, NM_001101.3; ABI ID, Hs99999903_m1; VIC-labeled] were purchased from Applied Biosystems (Foster City, CA, U.S.A.). PCR amplification and determination of the relative expression of specific genes were performed using an Applied Biosystems 7500 Sequence Detection System.

### Western blotting

Cells were washed with ice-cold phosphate buffered-saline. Total cell lysates were prepared using ice-cold RIPA cell lysis buffer (Thermo Scientific, Rockford, IL, U.S.A.) containing protease (Roche, Indianapolis, IN, U.S.A.) and phosphatase (Sigma) inhibitor cocktails. Protein concentrations were determined using the bicinchoninic acid assay (Pierce, Rockford, IL, U.S.A.). Equal amounts of proteins were subjected to SDS-PAGE and transferred to a nitrocellulose membrane (Millipore). The protein-blotted membrane was incubated in a blocking solution (10% skim milk) for 1 h at RT, followed by overnight incubation at 4°C in blocking solution containing a primary antibody. After 3 washes, the membrane was incubated for 40 min at RT with blocking solution containing an HRP-conjugated secondary antibody. The membrane was washed, and the immunoactive bands were visualized using an ECL (GE Healthcare, Piscataway, NJ, U.S.A.) or SuperSignal West Femto (Pierce) chemiluminescence substrate solution. Digital images were acquired using the Fusion-SL4 Spectra chemiluminescence system (Vilber, Eberhardzell, Germany). The primary and secondary antibodies employed in this study were as follows: rabbit anti-HIF-1α (Bethyl Laboratory, Montgomery, TX, U.S.A.), rabbit anti-HIF-2α (Abcam, Cambridge, MA, U.S.A.), rabbit anti-HIF-1β/ARNT(Cell Signaling Technology, Danvers, MA, U.S.A.), and β-actin (Bio-Rad, Hercules, CA, U.S.A.).

### Tumor-induced angiogenesis

**Figure 1:**
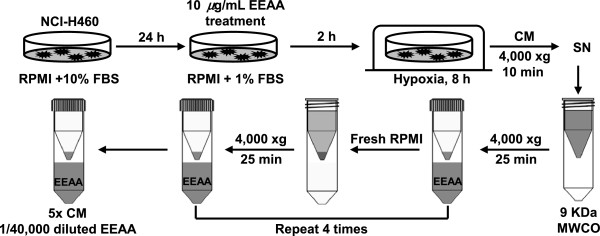
**Preparation of culture medium for tumor-induced angiogenesis.** Culture medium (CM) of NCI-H460 lung cancer cells was prepared as described in Methods section.

NCI-H460 cells were seeded at 3 × 10^6^ cells/dish in a 100 mm culture dish and then incubated for 24 h in complete growth medium. The cells were washed with serum-free RPMI basal medium and incubated for 2 h with basal medium supplemented with low serum (1% FBS) with or without 10 *μ*g/mL EEAA. Then, the cells were transferred to a hypoxic chamber filled with hypoxic gas (1% O_2_) for 8 h. The culture supernatant was harvested, and cell debris was spun down via centrifugation at 4,000 g for 10 min at 4°C. The cleared culture medium (SN) was concentrated using a 9 kDa protein concentrator (Pierce) at 4,000 g for 25 min at 4°C. The EEAA in the concentrated culture medium was diluted through 4 repeated solution exchanges with fresh basal medium (RPMI). The final 5-fold culture medium (5X CM) was saved and stored at −70°C until use. The overall process is illustrated in Figure 1. HUVECs were incubated in endothelial basal medium mixed with the same volume of 5-fold concentrated medium for cell proliferation, migration, and tube formation in vitro.

### Statistics

The differences in the means of continuous variables were determined using one-way analysis of variance and Tukey’s HSD post-hoc test. Statistical significance was set at *p* < 0.05.

## Results

### EEAA inhibits endothelial function-related angiogenesis in vitro and in vivo

**Figure 2:**
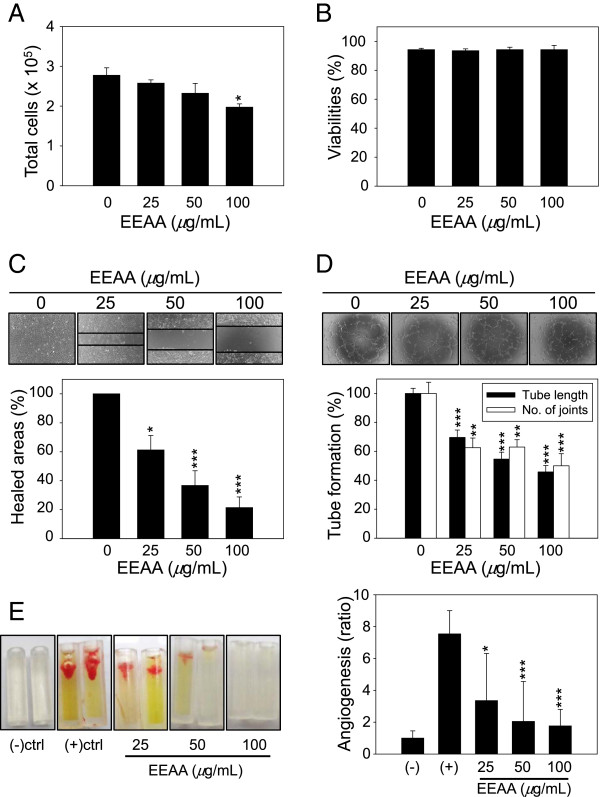
**EEAA inhibits angiogenesis in vitro and in vivo. (A and B)** Cell proliferation and viability of HUVECs after 24 h of EEAA treatment. Cells were treated with 25–100 *μ*g/mL EEAA for 24 h. Viable and dead cells were discriminated and counted using an automated cell counter, as described the Methods section. **(C)** Representative images depicting HUVEC migration after EEAA treatment with the indicated concentrations. Wound closures were measured and recorded at 0 and 18 h after injury. **(D)** Representative images depicting the formation of capillary-like tube structures on Matrigel by HUVECs after 8 h of EEAA treatment with the indicated concentrations. The effects of EEAA on the formation of capillary-like tube structures by HUVECs such as the changes in tube length and the number of joints, were quantified as described in the Methods section. The data are presented as the relative means ± S.D. of at least three independent experiments compared with the vehicle *P < 0.05, **P < 0.01, and ***P < 0.001 compared with the vehicle-treated groups. **(D and E) (E)** Effect of EEAA on angiogenic factor-derived vessel formation in vivo. Angioreactors filled with BME premixed with combinations of the angiogenic factors VEGF/FGF2 and various concentrations of EEAA (0–100 *μ*g/mL) were implanted into the dorsal flanks of nude mice. After 12 days, the implanted cylinders were harvested (left panel), and vascular endothelial cells that had migrated into the BME gels of the bioreactors were quantified using FITC-lectin detection (right panel). The negative control represents the bioreactors without VEGF/FGF2 inducers or EEAA. Relative angiogenesis was normalized to the mean of the negative control. *P < 0.05, and ***P < 0.001 compared with the positive control group.

The effects of EEAA on the angiogenic properties mediated by HUVECs were evaluated in vitro. EEAA efficiently inhibited HUVEC proliferation in a dose-dependent manner (Figure 2A) and a significant inhibitory effect of EEAA was observed at concentrations ≥ 100 *μ*g/mL. However, during the 24 h treatment period, no decrease in cell viability was observed and the cells maintained viabilities of ≥ 90% within this dosage range (0–100 *μ*g/mL) (Figure 2B). Therefore, the anti-proliferative effect of EEAA observed in HUVECs was not due to its cytotoxicity. EEAA dramatically inhibited HUVEC mobility at concentrations ≥ 25 *μ*g/mL, and only 21.4% of the original wound area was recovered by the HUVECs at 100 *μ*g/mL (Figure 2C). In addition HUVECs failed to form three dimensional tube like structure on the matrix supports in the presence of EEAA (Figure 2D). Image analysis revealed that EEAA significantly reduced tube length and the number of joints connecting tubes in a dose-dependent manner (Figure 2D).

Next, we investigated the effects of EEAA on the formation of new blood vessels in vivo. Vessel formation could not be observed in the absence of the pro-angiogenic inducers VEGF/FGF2 (negative control, Figure 2E). Massive vessel ingrowth was observed from the open ends of the angioreactors pre-filled with matrix gel mixed with VEGF/FGF2 (positive control), which was dramatically suppressed by EEAA in a dose-dependent manner (Figure 2E, left panel). Quantification of vessel formation by labeling endothelial cells with FITC-lectin revealed that significant anti-angiogenic effects of EEAA were observed at concentrations ≥ 25 *μ*g/mL, and vessel formation decreased to 23.3% in the 100 *μ*g/mL EEAA treatment compared with the positive control group (Figure 2E, right panel).

### EEAA down-regulates HIF signaling under hypoxia

**Figure 3:**
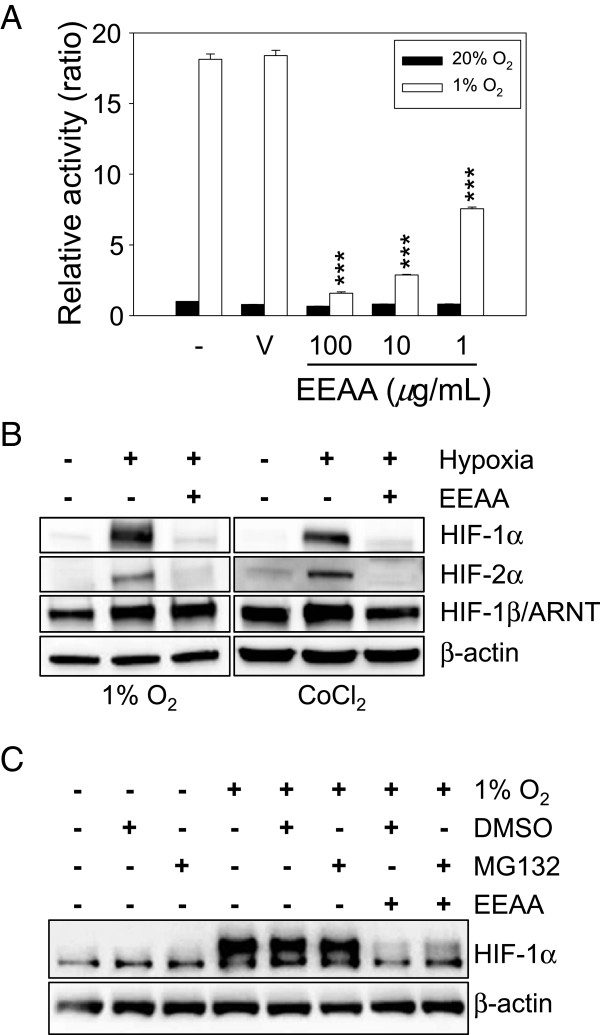
**EEAA down-regulates HIF signaling via enhanced proteasomal degradation. (A)** Effect of EEAA on HIF signaling. NIH3T3/HIF-luc cells were pre-treated with vehicle (DMSO) or with increasing concentrations of EEAA (1–100 *μ*g/mL) for 2 h before exposure to hypoxia (1% O_2_). After 24 h, intracellular luciferase activity was quantified. Relative luciferase activity was determined based on comparing with mock under normoxia (20% O_2_). The data are presented as the relative means ± S.D. of at least three independent experiments compared with vehicle. ***P < 0.001 compared with the vehicle groups. **(B)** Effect of EEAA on HIF protein expression under gas- or chemical-induced hypoxia. NCI-H460 cells were pre-treated with 100 *μ*g/mL EEAA for 2 h before exposure to hypoxic gas (1% O_2_) or to 150 *μ*M CoCl_2_. After 24 h of incubation, WCL was prepared and subjected to western blot analysis. **(C)** Enhanced proteasomal degradation of HIF-1α via EEAA. NCI-H460 cells were pre-treated with a combination of vehicle (DMSO) or 10 *μ*M MG132 and 100 *μ*g/mL EEAA for 2 h before exposure to hypoxic gas (1% O_2_). After 24 h of incubation, intracellular HIF-1α levels were determined through western blot analysis. β-actin was used as a loading control.

HIF signaling is known to play a pivotal role during tumor-induced angiogenesis and metastasis under hypoxic condition [18–20]. In this study, we evaluated the effect of EEAA on HIF signaling under hypoxic conditions using an NIH3T3 stable cell line (NIH3T3/HIF-luc) carrying 4 copies of HRE (5′-GTGACTACGTGCTGCCTAG-3′) sequences in the promoter region of the luciferase gene. As shown in Figure 3A, exposure of cells to hypoxia (1% O_2_) for 24 h increased the intracellular luciferase activity by 18-fold compared with the normoxia group (Figure 3A). However, EEAA down-regulated HIF signaling under hypoxia in a dose-dependent manner and more than 85% of luciferase activity was inhibited by 100 *μ*g/mL of EEAA.

To investigate the underlying mechanism of EEAA-mediated down-regulation of HIF signaling activity, intracellular level of HIF-1α/2α was determined using western blotting. As shown in Figure 3B, exposing NCI-H460 cells to 1% O_2_ (left panel) or to 150 *μ*M CoCl_2_ (right panel) for 24 h up-regulated the HIF-1α/2α proteins, which was markedly reversed by EEAA. HIF-1β/ARNT, which is known to be constitutively expressed, was not affected by hypoxic stress or by EEAA. Simultaneous treatment with MG132 (10 *μ*M), a proteasomal inhibitor, could partially relieve EEAA-mediated down-regulation of HIF-1α (Figure 3C), suggesting that EEAA can prevent HIF-1α accumulation under hypoxia by accelerating its proteasomal degradation.

### EEAA inhibits VEGF production in tumor cells

**Figure 4:**
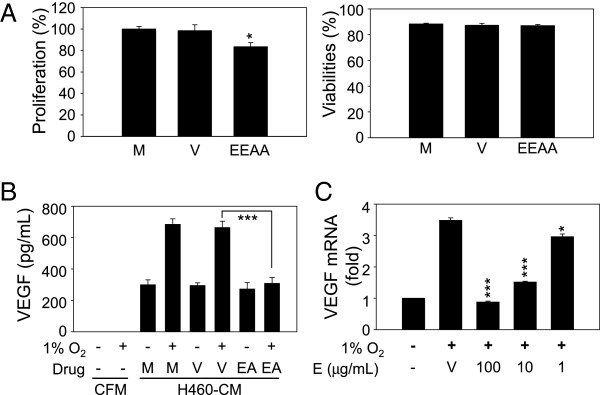
**EEAA decreases VEGF expression in tumor cells under hypoxic conditions. (A)** Effect of EEAA on tumor cell growth. HCI-H460 cells were treated with EEAA (10 *μ*g/mL), and cell proliferation (left panel) and viability were determined after 48 h. **(B)** Reduced production of VEGF due to EEAA. NCI-H460 cells were cultured under normoxic (20% O_2_) or hypoxic (1% O_2_) conditions in the absence (M) and the presence of vehicle (DMSO, V) or 10 *μ*g/mL EEAA (EA). After 8 h of incubation, an immunoassay was performed to quantify VEGF secreted from NCI-H460 cells. **(C)** Down-regulation of VEGF at the transcriptional level by EEAA. NCI-H460 cells were cultured under normoxic (20% O_2_) or hypoxic (1% O_2_) conditions in the presence of increasing concentrations of EEAA (1–100 *μ*g/mL). After 24 h, intracellular VEGF mRNA levels were quantified using RT-PCR. The relative mRNA contents were determined based on comparison with vehicle (DMSO). The data are presented as the mean or relative mean ± S.D. of at least three independent experiments. *P < 0.05 and ***P < 0.001 compared with the vehicle groups.

To examine the effects of EEAA on tumor-induced angiogenesis, we first determined the effect of EEAA on cell proliferation and viability in NCI-H460 tumor cells. EEAA treatment (10 *μ*g/mL) for 24 h inhibited cell proliferation by 16.5% compared with a mock (Figure 4A, left panel). However, cell viability was not affected by EEAA during this incubation time (Figure 4A, right panel). Next, we investigated whether EEAA could down-regulate VEGF expression in NCI-H460 cells under hypoxia. NCI-H460 cells were pre-treated with vehicle or with EEAA (10 *μ*g/mL) for 2 h and then incubated under hypoxia (1% O_2_) in RPMI 1640 medium supplemented with a low serum concentration (1%). After 8 h, VEGF released by the cells into the culture medium was quantified via ELISA. As shown in Figure 4B, hypoxia enhanced VEGF production in NCI-H460 cells by more than 2-fold compared with a normoxia group (M, (−) 1% O_2_). However, EEAA was able to reduce VEGF expression to the basal level (EA, (+) 1% O_2_). Vehicle did not affect hypoxia-induced VEGF expression (V, (+) 1% O_2_). The VEGF concentration in the cell-free medium (CFM) was too low to be detected, irrespective of hypoxia induction. To understand the mechanism underlying the down-regulation of VEGF expression by EEAA, we determined the alterations in intracellular levels of VEGF mRNA. Cells were pre-treated with the vehicle or EEAA for 2 h before exposure to hypoxia (1% O_2_). After 24 h, total RNA was isolated and subjected to quantitative RT-PCR. As shown in Figure 4C, VEGF mRNA levels decreased by EEAA in a dose-dependent manner, and the maximum EEAA treatment (100 *μ*g/mL) was able to reduce VEGF mRNA to the basal level.

### EEAA inhibits tumor-induced angiogenesis

**Figure 5:**
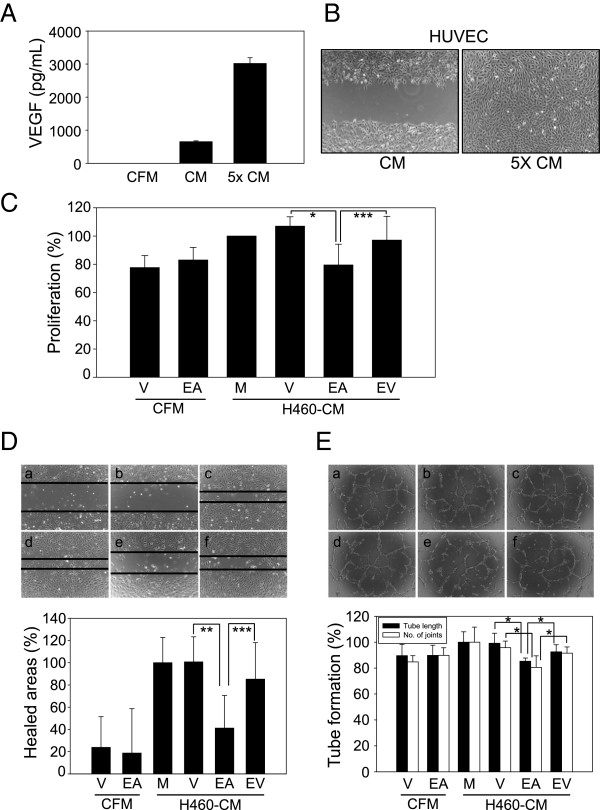
**EEAA suppresses tumor-induced angiogenesis by HUVECs in vitro. (A)** Concentration of tumor cell-culture medium. For NCI-H460 tumor cell-induced angiogenesis, the culture medium (CM) was concentrated 5-fold using a 9 kDa protein concentrator, as described in the Methods section and as depicted in Figure 1. The levels of VEGF in the cell-free medium (CFM), CM, and 5-fold CM (5X CM) were measured via ELISA. **(B)** Efficient removal of EEAA from the concentrated CM. HUVEC migration was observed in the presence of the CM or 5X CM. **(C to E)** The effect of EEAA on NCI-H460 tumor cell-induced angiogenesis mediated by HUVECs. The 5X CFM and 5X CM were prepared from cell-free (CFM) and NCI-H460 cells (H460-CM)-culture medium (CFM), respectively, in the absence (M) or the presence of vehicle (V), EEAA (EA), and VEGF supplementation (EV). Cell proliferation **(C)**, migration **(D)**, and tube formation **(E)** by HUVECs were determined after treatment with each concentrated culture medium. The data are presented as the relative mean ± S.D. of at least three independent experiments. *P < 0.05, **P < 0.01, and ***P < 0.001 compared with the vehicle groups.

The culture medium (CM) of NCI-H460 tumor cells was prepared as described in Figure 1. To minimize interference from the residual EEAA in the concentrated CM with assay, EEAA was depleted via 4 repeated solution exchanges with fresh RPMI basal medium. The efficacy of CM concentration and EEAA depletion in the 5X CM was confirmed by observing 4.6-fold increase in VEGF concentration in the 5X CM (Figure 5A) and intact cell migration in the presence of 5X CM (Figure 5B), respectively. We used this 5X CM for further experiments addressing in vitro tumor-induced angiogenesis.

The CM of NCI-H460 treated with mock (H460-CM-M) or vehicle (H460-CM-V) enhanced HUVEC proliferation (Figure 5C), cell mobility (Figure 5D), and three dimensional tube formation (Figure 5E). However, the EEAA-treated CM (H460-CM-EA) significantly inhibited these angiogenic properties mediated by HUVEC. Because EEAA was able to down-regulate the expression of VEGF under hypoxia (Figure 4B), we investigated whether the suppression of cell growth, migration, and tube formation via H460-CM-EA could be reversed by VEGF supplementation. As expected, H460-CM-EA supplemented with 3 ng/mL VEGF (H460-CM-EV) was able to relieve the inhibitory effects of EEAA on HUVEC-mediated angiogenic properties. These results indicate that EEAA prevented angiogenesis primarily by down-regulating VEGF production in NCI-H460 tumor cells.

## Discussion

Although massive and evident non-metastatic tumors can be eradicated through surgical intervention, the treatment of small primary tumors or cancers undergoing metastasis largely relies on non-invasive cancer therapies, such as irradiation and chemotherapy [6]. Because angiogenesis is an essential step during the development and metastasis of solid tumors, strategies targeting tumor angiogenesis for development of anticancer drugs have garnered much attention. Angiogenesis is a nearly universal characteristic of cancer, suggesting that anti-angiogenic drugs may present a wide range of clinical applications for cancer therapy [21]. Two pathways modulate angiogenesis, endothelium-related and tumor-induced angiogenesis pathways. Endothelium-related angiogenesis largely depends on modulating the ability of vascular endothelia cells to proliferate, migrate, and respond to pro-angiogenic proteins, such as VEGF, basic FGF (bFGF), interleukin 8, and transforming growth factor-β [6, 22]. Tumor-induced angiogenesis is based on the ability of tumors to modulate the expression and activity of pro-angiogenic proteins, such as VEGF and bFGF [6, 22], and is initiated by the release of these proteins from tumor cells, macrophages, and the extracellular matrix [23]. Pro-angiogenic factors recruit endothelial cells and promote their proliferation in preparation for blood vessel formation. In the present study, we showed that EEAA exhibits anti-angiogenic potential by regulating both endothelium-related (Figure 2) and tumor-induced angiogenic pathways (Figures 4 and 5).

Although we did not thoroughly investigate the mechanism, we suggested that EEAA can down-regulate VEGF expression under hypoxia by enhancing proteasomal degradation of its labile transcription factor, HIF-1/2α proteins. It is known that HIFα subunits hydroxylated by HIF-specific proly hydroxylases in the presence of O_2_ are subjected to degradation via the ubiquitin-proteasomal pathway [18, 24]. Previous studies demonstrating the mechanism of action of molecules inhibiting HIF-1α activities may provide some insight on this topic. Loss of the tumor suppressor protein, phosphatase and tensin homolog (PTEN) [25], and activation of the phosphatidylinositol 3-kinase (PI3K) pathway are related to increased levels of the HIF-1α protein [26]. PX-12 (1-methylpropyl 2-imidazolyl disulfide) decreases HIF-1α, independent of the von Hippel-Lindau (VHL) protein pathway, through irreversible inhibition of thioredoxin-1, which inhibits PTEN, leading to activation of the PI3K/Akt/mammalian target of rapamycin (mTOR) pathway [27, 28]. Rapamycin, which is an mTOR inhibitor, induces apoptotic cell death in human NSCLC cell lines through enhanced HIF-1α degradation and decreased HIF-1α − dependent expression of surviving protein under hypoxia [29]. YC-1 (3-(5′-hydroxymethyl-2′-furyl)-1-benzylindazole) down-regulates HIF-1/2α at both the transcriptional (protein synthesis) and post-transcriptional (protein stability) levels, which are linked to the metal ion-related pathway of O_2_ sensing and the murine double minute 2 protein [30–32]. As antagonists of heat shock protein 90 (hsp90), geldanamycin and 17-AAG (17-allylamino-17-desmethoxygeldanamycin) disrupt the physical interaction between hsp90 and HIF-1α and then promote proteasomal HIF-1α protein degradation, irrespective of VHL protein or O_2_ tension. A naturally occurring insecticide, deguelin, inhibits HIF-1α signaling by inhibiting de novo protein synthesis and promoting the proteasomal protein degradation of HIF-1α [33].

VEGF is a unique endothelial cell-specific mitogen that promotes many events required for angiogenesis, and is one of the important target genes regulated by HIF-1α [34]. HIF-1α, which is a component of a transcriptional complex that induces more than 60 genes required for tumors to adapt to hypoxic stress [31], is extremely labile under normoxia [35]. Under hypoxic conditions, HIF-1α accumulates and translocates to the nucleus, where this subunit interacts with HIF-1β, p300, and other transcription factors and then binds to HRE sequences residing in promoter regions of target proteins [36]. Because HIF-1α regulates a variety of processes, such as angiogenesis and glucose metabolism, this subunit is broadly accepted as a critically important tumor cell survival factor that is required for tumorigenesis and metastasis in early and late stages of tumors, respectively [37].

Some previous reports have demonstrated the anti-tumor effects of *A. atemoya* [14–16] and its parental plants, *A. squamosa* [38, 39] and *A. cherimola* [40]. However, to our knowledge, no previous study has verified the anti-angiogenic effect of an *A. atemoya* extract. Wu *et al*. reported that isodesacetyluvaricin, which is an annonaceous acetogenin, isolated from *A. glabra* inhibited the gene expression of cyclooxygenase-2 (COX-2), whose activity is associated with inflammation and with angiogenesis, in A431 human epidermoid carcinoma cells [41]. In the present study, we did not investigate the expression profile of COX-2 after EEAA treatment in A549 human lung cancer cells. However, the relationship between COX-2 and HIF-1α can be inferred from a previous study demonstrating that COX-2 can be transcriptionally up-regulated by HIF-1α under hypoxic conditions and that elevated COX-2 activity promotes the survival of colorectal tumor cells [42].

## Conclusions

Taken together, our findings demonstrated that EEAA exhibits anti-angiogenic potentials via direct inhibition of endothelial cell as well as tumor-mediated angiogenesis, including down-regulation of VEGF and HIF signaling in tumor cells. Therefore, EEAA should be considered a valuable herbal source for developing anticancer agents. The limitations of the present study include the followings: 1) we did not fully investigate the anti-angiogenic mechanisms of EEAA, such as down-regulation of HIF-1α under hypoxic conditions, and 2) we did not characterize the molecule(s) responsible for the observed EEAA-mediated anti-angiogenic potential.

## Authors’ information

Jin-Mu Yi is first author.

## Abbreviations

17-AAG: 17-allylamino-17-desmethoxygeldanamycin
bFGF: Basic fibroblast growth factor
BME: Basement membrane extract
CFM: Cell-free medium
CM: Culture medium
COX-2: Cyclooxygenase-2
DMSO: Dimethyl sulfoxide
EEAA: Ethanol extract of *Annona atemoya* seeds
FBS: Fetal bovine serum
FGF2: Fibroblast growth factor-2
HIF: Hypoxia-inducible factor
HRE: Hypoxia-responsive element
HRP: Horseradish peroxidase
Hsp90: Heat shock protein 90
HUVEC: Human umbilical vascular endothelial cells
KIOM: Korea Institute of Oriental Medicine
MDR: Multidrug-resistant
mTOR: Mammalian target of rapamycin
PI3K: Phosphatidylinositol 3-kinase
PTEN: Phosphatase and tensin homolog
PX-12: 1-methylpropyl 2-imidazolyl disulfide
RT: Room temperature
RT-PCR: Real-time polymerase chain reaction
VEGF: Vascular endothelial growth factor
VHL: Von Hippel-Lindau
YC-1: 3-(5′-hydroxymethyl-2′-furyl)-1-benzylindazole
WCL: Whole cell .
